# In vitro Study of the Effects of Acetylcysteine on the International Normalized Ratio Over Time

**DOI:** 10.1097/FTD.0000000000001356

**Published:** 2025-07-14

**Authors:** Messia Nazar, Jenny E. Kootstra-Ros, Daniel J. Touw, Marieke G.G. Sturkenboom

**Affiliations:** * Department of Clinical Pharmacy and Pharmacology, University of Groningen, University Medical Center Groningen, Groningen, the Netherlands;; † Department of Laboratory Medicine, Groningen, University of Groningen, University Medical Center Groningen, the Netherlands; and; ‡ Department of Pharmaceutical Analysis, University of Groningen, Groningen Research Institute of Pharmacy, Groningen, the Netherlands.

**Keywords:** acetylcysteine, international normalized ratio, coagulation, prothrombin time

## Abstract

**Background:**

Patients with paracetamol poisoning are treated with acetylcysteine. In paracetamol-poisoned patients without hepatocellular injury, an increased international normalized ratio (INR) was observed after acetylcysteine administration. Because INR is a marker of liver injury in paracetamol poisoning, assessing the magnitude of the effect of acetylcysteine treatment on INR is crucial. This in vitro study aimed to quantify the effects of clinically relevant concentrations of acetylcysteine on INR over time.

**Methods:**

Acetylcysteine (0–500 mg/L) in Omniplasma (pooled plasma) or citrate plasma from 6 healthy subjects was incubated at 37°C for up to 24 or 6 hours, respectively. Combined with acetylcysteine, the effects of paracetamol (0–200 mg/L), incubation temperature (37, 20, and 4°C), and tube size (15 and 2.5 mL) were investigated. Samples were drawn frequently and analyzed for INR. Citrate plasma from healthy subjects was used to assess interindividual variability. Data were analyzed using a generalized linear mixed model.

**Results:**

At 37°C, every mmol/L (equals 163 mg/L) of acetylcysteine increased the INR with 0.039 (99% confidence interval [CI] 0.032–0.046, *P* < 0.0001), whereas every mmol/L (equals 151 mg/L) of paracetamol increased the INR with 0.028 (99% CI 0.013–0.044, *P* < 0.001). Independent of acetylcysteine and paracetamol, every hour of incubation at 37°C increased the INR by 0.012 (99% CI 0.011–0.014, *P* < 0.0001). Incubating 100, 200, and 500 mg/L acetylcysteine in plasma from healthy subjects at 37°C showed large interindividual variability in INR from 0.058 (99% CI 0.029–0.086, *P* < 0.001) to 0.173 (99% CI 0.131–0.216, *P* < 0.0001).

**Conclusions:**

This in vitro study showed a clinically significant effect of increasing acetylcysteine concentrations on INR over time at 37°C in pooled plasma. Additionally, a large interindividual variability was observed.

## Abstract 2

Supplemental Digital Content is Available in the Text.

## INTRODUCTION

Paracetamol (acetaminophen, N-acetyl para-aminophenol, or APAP) is one of the most widely used analgesics. However, paracetamol is frequently overdosed due to its availability, either deliberately or unintentionally.^1^ In paracetamol poisoning, normal metabolic pathways such as glucuronidation and sulfation become saturated, and the hepatotoxic metabolite N-acetyl-para-benzoquinone imine (NAPQI) is synthesized.^2^ The most important prognostic markers for hepatotoxicity are increased liver enzyme concentrations (alanine transaminase (ALT) or aspartate aminotransferase >1000 IU/L), prolonged prothrombin time (PT)/increased international normalized ratio (INR), and prolonged paracetamol half-life (>4 hours).^3,4^

Patients with paracetamol poisoning are treated with the antidote acetylcysteine.^3^ As an inorganic sulfate source, acetylcysteine stimulates paracetamol metabolism through the sulfation pathway.^5^ More importantly, it induces the glutathione synthesis to enhance detoxification by forming adducts with NAPQI.^3,5^ Acetylcysteine treatment is initiated when the paracetamol concentration is above the Rumack-Matthew nomogram.^3^ There are different acetylcysteine regimens available, most delivering 300 mg/kg body weight of acetylcysteine over 12–21 hours^3,6^ In the Scottish and Newcastle Anti-emetic Pre-treatment for paracetamol poisoning (SNAP) regimen, a loading dose of 100 mg/kg acetylcysteine is infused over 2 hours, followed by a maintenance dose of 200 mg/kg over 10 hours^3,6^ Paracetamol concentration, ALT, and INR are measured before and after 10 hours of acetylcysteine treatment.^6^ If at 10 hours, paracetamol concentration is <20 mg/L, ALT <100 IU/L, not more than doubled from admission ALT, and INR ≤1.3, treatment is stopped after the maintenance dose has been administered.^6^ If these criteria are not met, acetylcysteine treatment is continued at 200 mg/kg over 10 hours^6^

In healthy subjects, acetylcysteine increases the PT/INR by decreasing the activity of clotting factors II, VII, IX, and X.^7^ This effect of acetylcysteine on PT/INR was also observed in patients with paracetamol poisoning without hepatocellular injury.^7–9^ In vitro incubation of plasma from healthy subjects with 550 mg/L acetylcysteine significantly increases the INR by a mean of 17%.^10^ However, the effects of time were not investigated in this study. Additionally, the acetylcysteine concentrations were higher than expected when using the SNAP regimen. Monte Carlo simulations of the SNAP regimen estimated a mean peak plasma acetylcysteine concentration of 300 mg/L (95% confidence interval [CI] 200–400 mg/L) at the end of the loading dose, which decreased to 220 mg/L (95% CI 75–400 mg/L) at 12 hours^11^

Paracetamol itself also increases INR by affecting factor VII activity.^12,13^ Owens et al*^12^* developed a pharmacokinetic–pharmacodynamic model that described the effects of paracetamol and acetylcysteine on the INR. For paracetamol, the maximum effect was estimated to be 0.534 (increase from baseline INR) with 197 mg/L paracetamol, yielding 50% of this effect (EC_50_). The maximum effect was 0.325 for acetylcysteine. The authors were unable to estimate the EC_50_ of acetylcysteine because concentration data were unavailable.

Therefore, this in vitro study aimed to quantify the effect of clinically relevant acetylcysteine concentrations on INR after incubation at 37°C for a maximum of 24 hours.

## MATERIALS AND METHODS

Acetylcysteine concentrations from 0 to 500 mg/L in pooled plasma (Omniplasma, Octopharma, Langenfeld, Germany) or citrate plasma from healthy subjects were incubated at 37°C for up to 24 hours and 6 h, respectively (see **Supplementary File 1**, **Supplemental Digital Content 1**, http://links.lww.com/TDM/A863). A 24-hour period was selected to account for the 2 maintenance doses in the SNAP regimen and to align with other 21-hour acetylcysteine protocols. The effects of different paracetamol concentrations, incubation temperatures, incubation volumes/tube sizes, and opening/closing of incubation tubes were investigated (Table 1). This study was performed at the Departments of Clinical Pharmacy and Pharmacology and Laboratory Medicine of the University Medical Center Groningen, Groningen, the Netherlands.

**TABLE 1.** Variables Investigated in the In vitro Experiments

Variable	Options
Incubation time	0, 0.25, 0.5, 0.75, 1.0, 1.5, 2.0, 2.5, 3.0, 3.5, 4.0, 4.5, 5.0, 5.5, 6.0, 8, 10, 12, 16, 20, and 24 h
Acetylcysteine concentration	0, 25, 50, 100, 200, 300, 400, and 500 mg/L
Paracetamol concentration	0, 50, 100, 150, and 200 mg/L
Plasma origin	Pooled plasma (Omniplasma), citrate plasma from 6 healthy subjects, and pooled plasma from these subjects
Incubation temperature	4, 20, and 37°C
Tube size	2.5 and 15 mL
Opening of tube	Single and multiple times opening of tubes

A test run was performed wherein 3 acetylcysteine concentrations (0, 25, and 500 mg/L) in citrate plasma from a healthy subject were incubated in 2.5-mL tubes. Samples drawn at 0, 2, 12, and 24 hours after incubation at 37°C were analyzed for PT/INR. The experimental protocol was not modified in the test run; therefore, data from the test run were used for data analysis.

Plasma samples from different sources were used in this study. Most of the tests were performed using pooled plasma (Omniplasma). Omniplasma is pooled human plasma derived from 600 to 1200 healthy subjects.^14^ Plasma was pooled to ensure consistent concentrations of coagulation factors and other proteins per Omniplasma batch.^15^ Before being used in the experiments, Omniplasma was thawed in a water bath according to product instructions.^15^

Acetylcysteine (Fluimucil 200 mg/mL, Zambon, Amersfoort, the Netherlands) was diluted with water for injection (Braun, Melsungen, Germany). The acetylcysteine stock solutions were prepared daily. To ensure consistency across the study, different concentrations of acetylcysteine stock solutions were spiked to pooled plasma (Omniplasma) in a constant ratio of 1:200 in 15-mL tubes (Sarstedt, Numbrecht, Germany). The final plasma acetylcysteine concentrations were 0, 25, 50, 100, 200, 300, 400, and 500 mg/L. The concentration range was based on the 95% CI concentrations simulated for the SNAP regimen.^11^ Pooled plasma (Omniplasma) with acetylcysteine was incubated in a water bath (VWR, Amsterdam, the Netherlands) at 37°C for up to 24 hours. To investigate the effect of incubation temperature, additional 0 and 200 mg/L of acetylcysteine in pooled plasma were incubated for 24 hours at room temperature (20°C) and 4°C. Sampling was performed at 0, 1, 2, 3, 4, 5, 6, 8, 10, 12, 16, 20, and 24 hours postincubation. To investigate the development of the effects in the first 6 hours of incubation at 37°C more closely, an additional test was performed with 0, 100, 200, and 500 mg/L of acetylcysteine in pooled plasma (Omniplasma). Sampling was performed at 0, 0.25, 0.5, 0.75, 1.0, 1.5, 2.0, 2.5, 3.0, 3.5, 4.0, 4.5, 5.0, 5.5, and 6.0 hours postincubation.

Paracetamol 10 mg/mL of injection (Fresenius Kabi, Huis ter Heide, Netherlands) was diluted with water for injection into different concentrations of paracetamol stock solution. Different paracetamol stock solutions were spiked to pooled plasma (Omniplasma) in a constant ratio of 1:50; this lower ratio was due to the lower concentration of the paracetamol injection. The final concentration of paracetamol in the pooled plasma ranged from 0 to 200 mg/L (0, 50, 100, 150, and 200 mg/L). These concentrations were chosen because they represent clinically relevant paracetamol concentrations in patients with paracetamol poisoning, and the upper range reflects the EC_50_ as reported by Owens et al.^12^ Because we were unable to simulate paracetamol elimination in this in vitro test, its concentration remained stable throughout the experiment. The acetylcysteine concentrations investigated in the combined tests were 0, 100, 200, and 500 mg/L. All combinations of acetylcysteine and paracetamol concentrations were tested. Sampling was performed at 0, 1, 2, 3, 4, and 6 hours postincubation at 37°C.

To investigate the interindividual variability in INR, citrate plasma from 6 healthy subjects was incubated with acetylcysteine at 37°C up to 6 hours. In addition, pooled plasma samples from 6 healthy subjects were tested. Before blood was drawn, all the subjects were judged to be healthy. Written informed consent was obtained from all subjects. The requirement for ethical clearance was waived by the Medical Ethical Review Board University Medical Center Groningen (M24.343,573). Venous blood was obtained from each participant with minimal stasis using a tourniquet. Samples were collected in tubes containing 3.2% sodium citrate (Greiner Bio-One; Greiner, Kremsmünster, Austria). The tubes were centrifuged at 1880*g* for 5 minutes and used directly for the experiment. The investigated concentrations in the interindividual variability test were 0, 100, 200, and 500 mg/L acetylcysteine in plasma, and sampling was performed at 0, 1, 2, 4, and 6 hours postincubation at 37°C.

The effect of incubation volume and opening and closing of tubes was investigated using 2.5-mL test tubes (Sarstedt, Germany) and 2 sets in 15-mL test tubes. One set of 15-mL tubes was shaken, opened, sampled, and closed at every sampling time. The second set was shaken, opened, and sampled during the sampling time. The investigated concentrations in this test were 0, 200, and 500 mg/L acetylcysteine in pooled plasma, and sampling was performed at 0, 16, 20, and 24 hours postincubation at 37°C.

Immediately after every sampling time point in all experiments, samples were analyzed for PT/INR using ACL TOP550CTS (Werfen, Breda, the Netherlands) and HemosIL RecombiPlasTin 2 G (Werfen, Breda, the Netherlands) as reagent. The PT range was 10–150 seconds, and the correlation variances were 0.8% and 2.2% at 11 and 22 seconds, respectively.

Because of the longitudinal data and different variables (Table 1), a generalized linear mixed-model (GLMM) analysis was performed. All investigated variables (acetylcysteine concentration, paracetamol concentration, incubation time, plasma origin, temperature, tube size, and single/multiple openings) were independent of each other. In the GLMM, the concentrations of acetylcysteine and paracetamol were converted to mmol/L, corresponding to 163 and 151 mg/L, respectively. For the nominal variables, the option with the largest numbers of results was chosen as reference: 37°C, pooled plasma (Omniplasma), and 15-mL tube and multiple times opened. GLMM was performed using SPSS version 28.0 (IBM, Armonk, NY). Statistical significance was set at *P* < 0.05. Graphs were constructed using PyPlot 3.9.1. Python.

## RESULTS

**FIGURE 1.**
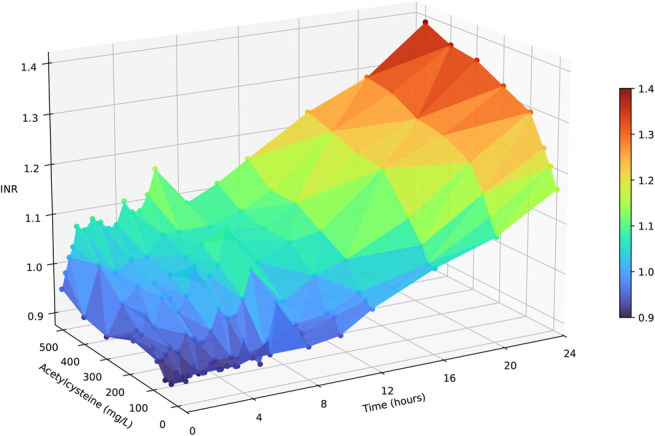
Effect of acetylcysteine concentration and incubation time on INR in pooled plasma (Omniplasma) at 37°C.

**FIGURE 2.**
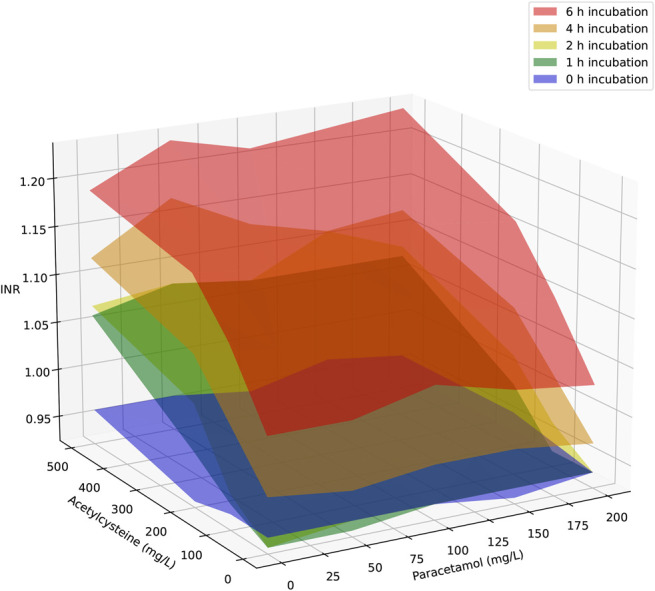
Effect of acetylcysteine and paracetamol concentration on INR in pooled plasma (Omniplasma) at 37°C at 0, 1, 2, 4, and 6 hours after incubation. No incubation (0 hours): blue, 1-hour incubation: green, 2-hour incubation: yellow, 4-hour incubation: orange, 6-hour incubation: red.

A total of 559 samples were analyzed for PT/INR. Owing to the differences in experiments and sampling times, the number of measurements per acetylcysteine concentration and incubation time varied. At 37°C in pooled plasma (Omniplasma), 239 results were obtained: most combinations of acetylcysteine concentration and incubation time were tested once (n = 99). Thirteen combinations were tested 4 times, 10 were tested 3 times, 7 were tested 2 times, 6 were tested 6 times, and 1 combination (0 mg/L at 0 hours) was tested 8 times. When multiple measurements were available for the same concentration and incubation time, their mean was used in Figure 1. Figure 1 illustrates the effect of acetylcysteine concentration and incubation time on INR in pooled plasma (Omniplasma) at 37°C. As shown, INR increases with both higher acetylcysteine concentrations and longer incubation times. Figure 2 shows the combined effect of acetylcysteine and paracetamol on INR across different incubation periods. The effect of paracetamol was less pronounced than that of acetylcysteine, partly due to the lower concentrations tested.

**FIGURE 3.**
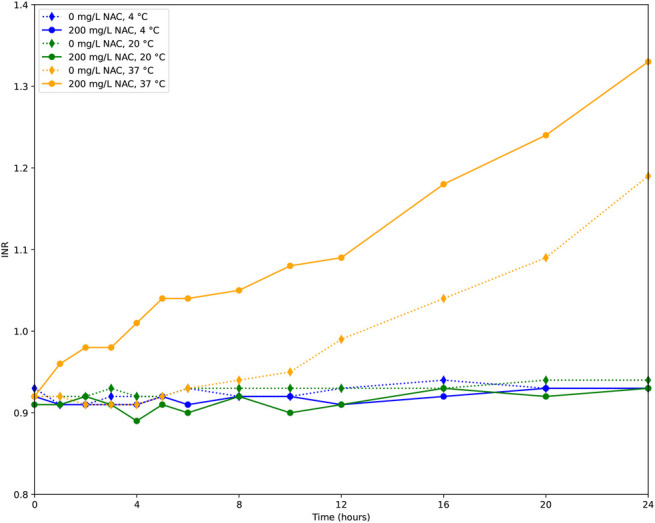
Influence of incubation temperature and time on INR at 0 and 200 mg/L acetylcysteine. 4°C: blue; 20°C: green; 37°C: yellow/orange; 0 mg/L acetylcysteine dashed line with diamonds; 200 mg/L acetylcysteine solid line with circles.

The results of the GLMM analysis are presented in Table 2. At 37°C, 1 mmol/L (equals 163 mg/L) acetylcysteine increased the INR by 0.039 (*P* < 0.0001). The INR increased by 0.028 (*P* < 0.001) for every mmol/L (151 mg/L) of paracetamol. The INR increased every hour of incubation at 37°C by 0.012 (*P* < 0.0001, Table 2). The INR remained at baseline for both 0 and 200 mg/L of acetylcysteine, when incubation was performed at 20 or at 4°C (Fig. 3). Incubation in the smaller tube led to an additional increase in the INR of 0.054 (*P* = 0.036). No difference was observed between the tubes that were opened once or multiple times (Table 2).

**TABLE 2.** Effect of Different Variables on the Change in INR

	Coefficient	99% CI	*P*
Acetylcysteine (mmol/L, 163 mg/L)	0.039	0.032 to 0.046	<0.0001
Paracetamol (mmol/L, 151 mg/L)	0.028	0.013 to 0.044	<0.001
Time (h)	0.012	0.011 to 0.014	<0.0001
Incubation temperature			
37°C	*Reference*		
Room temperature (20°C)	−0.121	−0.166 to −0.077	<0.001
4°C	−0.121	−0.165 to −0.077	<0.001
Tube size			
15 mL	*Reference*		
2.5 mL	0.054	−0.012 to (+)0.121	0.036
Opening/closing			
Opened multiple times	*Reference*		
Opened single time	−0.017	−0.064 to (+)0.033	0.351
Plasma origin			
Omniplasma	*Reference*		
Pooled S1–S6	0.113	0.070 to 0.156	<0.001
Subject 1	0.136	0.095 to 0.178	<0.001
Subject 2	0.067	0.028 to 0.106	<0.001
Subject 3	0.149	0.111 to 0.188	<0.0001
Subject 4	0.124	0.068 to 0.179	<0.001
Subject 5	0.173	0.131 to 0.216	<0.0001
Subject 6	0.058	0.029 to 0.086	<0.001

99% CI, 99% confidence interval.

Regardless of acetylcysteine concentration or incubation time, substantial interindividual variability in INR increase was observed, ranging from 0.058 to 0.173 compared with that of Omniplasma (*P* < 0.001, Table 2). Acetylcysteine incubated in pooled plasma from the 6 subjects resulted in an additional INR increase of 0.113 compared with incubation in Omniplasma (*P* < 0.001).

## DISCUSSION

This in vitro study showed a small but significant increase in the INR with increasing acetylcysteine concentrations over time. The concentration-dependent effect of acetylcysteine on INR has been reported previously, both in vitro and in healthy subjects, as well as in patients with paracetamol poisoning.^7^ As shown in Figure 1, the largest increase in the INR occurred within the first 6 hours of incubation. This mirrors an in vivo study where 27% maximum decrease in combined factor II + VII + IX activity was observed after 6–8 hours following a 400 mg/kg acetylcysteine infusion over 36 hours in healthy subjects.^16^ At higher supratherapeutic concentrations (5 mmol/L or 815 mg/L), acetylcysteine caused a similar maximum decrease (30%) in combined factor II + VII + IX activity, but this occurred within just 1 hour and remained reduced throughout 6 hours of incubation at 37°C.^17^

Notably, plasma incubated without acetylcysteine at 37°C also showed a significant increase in INR over time, unlike at 4°C and 20°C (Figs. 1 and 2). This may be due to clotting factor instability in vitro or the consumption of clotting factors during incubation at physiological temperature. Because no fresh plasma or coagulation factors are supplied in vitro*,* clotting factor activity declines and remains low over time. This phenomenon was not observed in Thorsen et al*^17^* study, likely because their incubation period was limited to 6 hours, and they did not include a no-acetylcysteine control.

At 20°C and 4°C, 200 mg/L of acetylcysteine had no effect on INR over time (Fig. 2). This agrees with Thorsen et al,^17^ where the supratherapeutic concentration of 5 mmol/L (815 mg/L) acetylcysteine caused only minor reductions (10% and 5%) in combined factor II + VII + IX activity at 24°C and 4°C, respectively. The reduced enzymatic activity of coagulation factors at temperatures below 33°C likely explains this finding.^18^ Additionally, cold storage (2–8°C) may lead to factor VII activation, which could further influence results.^19^

In our study, 1 mmol/L (151 mg/L) paracetamol increased the INR by 0.028. This increase was very small compared with the modelled 50% maximum effect, 0.267, at 197 mg/L paracetamol as EC_50_.^12^ This difference may be partly explained by the fact that massive overdoses (up to 75 g) of paracetamol were included, and the INR increase may have been caused by hepatic injury.^12^ The effect of paracetamol on INR was also shown to be time dependent, with increased INR being more common at 12 hours or more postingestion.^13^ In this in vitro study, we measured the effect only up to a concentration of 200 mg/L (1.3 mmol/L) for up to 6 hours postincubation. Finally, it has been shown in vitro that the anticoagulant effect of paracetamol is caused by the irreversible inactivation of vitamin K by NAPQI.^20^ This effect already occurs in the micromolar range of NAPQI and may arise after therapeutic doses of paracetamol.^20^ By contrast, we confirmed that the maximum effect of 0.325 acetylcysteine, which was modelled by Owens et al*,*^12^ could be achieved with higher concentrations of acetylcysteine and/or longer incubation periods.

Large interindividual variability in INR was observed when acetylcysteine was incubated in plasma from 6 healthy subjects, ranging from 0.058 to 0.173 compared with pooled plasma (Omniplasma) as the reference matrix (Table 2). This indicates that individuals react differently to the effect of acetylcysteine on the INR and that the observed effect of acetylcysteine in Omniplasma may be underestimated in specific individuals.

The INR increased significantly more in the small tubes (2.5 mL) than that in the large 15-mL tubes (Table 2). The rationale behind this finding is unclear; however, we hypothesized that fluctuations in temperature in smaller volumes during incubation in the water bath may play a role.

This study has some limitations: Most data (407/559 results) were obtained using pooled plasma (Omniplasma) that was standardized to the concentration of coagulation factors, possibly underestimating the effect of acetylcysteine. To estimate the difference from pooled plasma, a test was conducted using plasma from healthy subjects, which revealed substantial interindividual variability. More importantly, in the in vitro system, neither acetylcysteine nor paracetamol was metabolized, and the effects of their metabolites were not examined. Therefore, caution is warranted when translating these in vitro findings into clinical practice.

Although the in vitro effect of acetylcysteine on the INR appears modest, our results show that INR continues to rise during incubation. This is consistent with previous studies demonstrating that PT, INR, and/or clotting factor activity only return to baseline after discontinuation of acetylcysteine.^7^ This finding is particularly relevant for hospitals using the SNAP regimen, where acetylcysteine treatment should be continued if the INR exceeds 1.3.^6^ Applying our pooled plasma (Omniplasma) data to the estimated mean (95% CI) plasma concentrations achieved with the SNAP regimen suggests an INR increase of 0.17 (0.14–0.22) after 10 hours of treatment. Although this may seem modest, it is important to consider interindividual variability, which in our healthy subjects ranged from 0.058 to 0.173. A total INR increase of 0.2–0.4 aligns with observations in clinical practice and supports the effect of 0.325 modeled by Owens et al.^12^

Our in vitro study suggests that clinically relevant acetylcysteine concentrations increase INR over time. Previous studies have shown that in vitro increases in the INR are comparable to those observed in vivo.^7^ Next, a similar increase of 15%–30% increase in PT/INR was observed in patients with paracetamol poisoning,^7–9,21^ indicating that these data may reflect those of clinical practice in patients with paracetamol poisoning. These in vitro data confirm that an isolated increase in INR during acetylcysteine infusion does not necessarily reflect liver dysfunction.^7^

## CONCLUSION

Increasing concentrations of acetylcysteine have a clinically significant effect on INR over time at 37°C. This effect should be considered when a solitary increase in INR is observed during acetylcysteine treatment.

## Supplementary Material
