# Supplementary figures and images for: In vitro Study of the Effects of Acetylcysteine on the International Normalized Ratio Over Time
Source: Ther Drug Monit. 2025 Jul 14;48(3):393–9. doi: 10.1097/FTD.0000000000001356 (PMC13152081; doi:10.1097/FTD.0000000000001356)

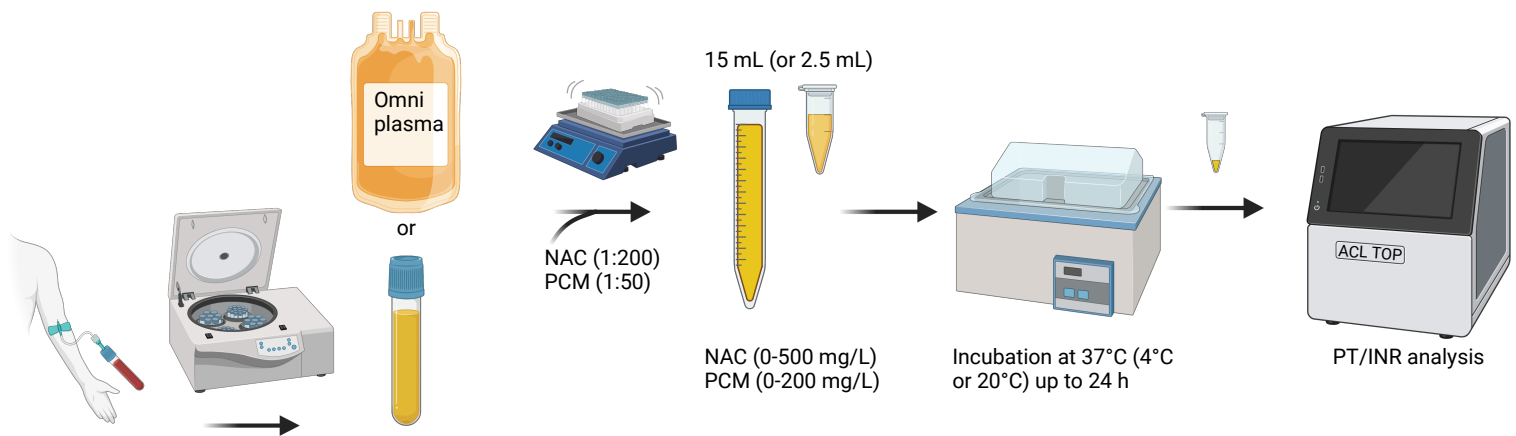

Supplement: Supplementary file 1 [file tdm-48-393-s001.pdf]
