# Supplementary material for: In vitro Study of the Effects of Acetylcysteine on the International Normalized Ratio Over Time
Source: Ther Drug Monit. 2025 Jul 14;48(3):393–9. doi: 10.1097/FTD.0000000000001356 (PMC13152081; doi:10.1097/FTD.0000000000001356)
Supplement: Supplementary file 2 [file tdm-48-393-s002.pdf]

Dear Author,

Please review the fees to produce your figures in color in *Therapeutic Drug Monitoring*. The price for the first color figure is $750. The charge for each additional color figure is $150.

If this charge meets with your approval, please identify which figures/pieces should be printed in color, sign below, and email a copy of this letter to my attention. If your institution or affiliation will cover the cost, please have your purchasing agent sign below. You will be invoiced for all article charges (color separations, reprints, etc.) shortly after your proof corrections are returned.

If you do not wish to have your figures print in color, please indicate "Decline Color" below. The figures will appear in black and white in print, and in color in the online version of your article. There is no charge for this service.

Prompt return of the signed form, even if you choose to decline color, will avoid delays in publication.

Thank you.

The Proof Manager
Health Learning, Research & Practice
Wolters Kluwer Health
Email: lww@tnq.co.in

Article Number: TDM25-077(R1)

Figures to appear in color: in print none, online 3 figures in color

Total cost for color figures: 0 (zero) USD

■ Decline Color

Marieke G.G. Sturkenboom

Printed Name

Marieke Sturkenboom

Digitaal ondertekend door Marieke Sturkenboom
Datum: 2025.06.23 22:15:14 +02'00'

Signature

June 23, 2025

Date
